# Pacific Walrus (*Odobenus rosmarus divergens*) Resource Selection in the Northern Bering Sea

**DOI:** 10.1371/journal.pone.0093035

**Published:** 2014-04-09

**Authors:** Chadwick V. Jay, Jacqueline M. Grebmeier, Anthony S. Fischbach, Trent L. McDonald, Lee W. Cooper, Fawn Hornsby

**Affiliations:** 1 Alaska Science Center, U.S. Geological Survey, Anchorage, Alaska, United States of America; 2 Chesapeake Biological Laboratory, University of Maryland Center for Environmental Science, Solomons, Maryland, United States of America; 3 Western EcoSystems Technology, Inc., Laramie, Wyoming, United States of America; Université du Québec à Rimouski, Canada

## Abstract

The Pacific walrus is a large benthivore with an annual range extending across the continental shelves of the Bering and Chukchi Seas. We used a discrete choice model to estimate site selection by adult radio-tagged walruses relative to the availability of the caloric biomass of benthic infauna and sea ice concentration in a prominent walrus wintering area in the northern Bering Sea (St. Lawrence Island polynya) in 2006, 2008, and 2009. At least 60% of the total caloric biomass of dominant macroinfauna in the study area was composed of members of the bivalve families Nuculidae, Tellinidae, and Nuculanidae. Model estimates indicated walrus site selection was related most strongly to tellinid bivalve caloric biomass distribution and that walruses selected lower ice concentrations from the mostly high ice concentrations that were available to them (quartiles: 76%, 93%, and 99%). Areas with high average predicted walrus site selection generally coincided with areas of high organic carbon input identified in other studies. Projected decreases in sea ice in the St. Lawrence Island polynya and the potential for a concomitant decline of bivalves in the region could result in a northward shift in the wintering grounds of walruses in the northern Bering Sea.

## Introduction

Knowledge of how a population selects resources enables a better understanding of how changes in the availability of those resources may affect the population's distribution and abundance. Evidence of selection is the use of a resource at a level that is disproportionate to the resource's availability. Many factors can contribute to selection, including competition, predator density, prey density and quality, and spatial patterns of habitat [1].

Arctic marine mammals are experiencing substantial changes to their sea ice habitat. The potential negative effects of sea ice loss to marine mammals have been recognized [2], [3], [4], including recent observations of changes in Pacific walrus areas of use in response to sparse sea ice [5] and projections of worsening sea ice conditions in coming decades [6].

Walruses require a substrate to rest upon between foraging trips, and when possible, adult females and young use sea ice throughout their seasonal ranges [7]. However, the persistence and extent of the sea ice habitat of the Pacific walrus is undergoing rapid change from climate warming. Although the loss of sea ice habitat is most acute in summer and fall in the Chukchi Sea [6], the southern boundary of sea ice in the Bering sea is projected to shift northward during the 21^st^ century [8], [9].

Heterogeneity or patchiness of resources occurs over multiple temporal and spatial scales [10], [11], [12], [13] and an animal's selection of resources can be thought of as a hierarchical process [14], [15]. Selection hierarchy can be defined wherein a species' first-order selection is the choice of geographic range, second-order selection is the choice of home ranges within the geographic range, third-order selection is the choice of habitat components within a home range, and fourth-order selection is the choice of specific resources within a habitat component [15].

The geographic range of the Pacific walrus (*Odobenus rosmarus divergens*) (i.e., first-order selection) occurs over the continental shelf, mainly within the Chukchi Sea from the eastern East Siberian Sea to western Beaufort Sea and the Bering Sea from Kamchatka to Bristol Bay [16]. A very small population of walruses occupying the Laptev Sea, and separated from the Chukchi Sea walruses by the East Siberian Sea, was recently assigned to the Pacific subspecies (*O. r. divergens*) [17]. Pacific walruses forage on the seafloor, primarily for infaunal invertebrates, and the continental shelves of the Chukchi and Bering Seas provide extensive areas with high benthic biomass [18], [19], [20] and shallow waters where diving to the seafloor is energetically feasible [21]. Walruses consume a wide range of organisms, from small crustaceans to seals [7], but their predominant prey in the Chukchi and Bering Seas are bivalves, gastropods, and polychaetes [22].

The seasonal home range of the Pacific walrus (i.e., second-order selection) is delimited by the continental shelves of the Chukchi and Bering Seas in summer and the Bering Sea in winter. In winter, walruses form three main breeding concentrations: a small concentration in the western Bering Sea (Anadyr Gulf in Russia), a moderate concentration in the southeastern Bering Sea (from Nunivak and the Pribilof Islands to Bristol Bay), and a large concentration in the northern Bering Sea [7].

The northern Bering Sea walrus concentration occurs in the region of the St. Lawrence Island polynya (cf. “St. Lawrence Island Polynya, South” in Stringer and Groves [23]). The St. Lawrence Island winter polynya typically extends ∼25 km southward of the island or further depending upon local winds [24]. Hydrographic studies indicate that locally high nutrient concentrations and phytoplankton production results in a high deposition of organic carbon to the benthos [25], [26], [27], [28]. Benthic biomass in the region of the St. Lawrence Island polynya is enhanced immediately south of the island by rich nutrients from an eastward flowing branch of the Anadyr Current, and to the southwest of the polynya by hydrographic accumulation of phytodetritus from water-column production over a larger area [25], [29].

There are few data on the diet of walruses specific to the region of the St. Lawrence Island polynya [22], [30]. Macroinfauna in the region is dominated by bivalves, amphipods, and polychaetes [18]; however, a decadal shift in species dominance has occurred [20], with substantial changes to the distribution and abundance of some bivalve species within only a few years [31]. Some evidence suggests the northern Bering Sea is exhibiting a trend away from extensive seasonal sea ice cover and high benthic production toward more subarctic ecosystem conditions favoring pelagic species, which could have a large effect on the future availability of benthic prey for walruses [19], [32].

We investigated walrus selection of benthic macroinfauna and sea ice in the region of the St. Lawrence Island polynya (i.e., third-order selection) using a discrete choice model [33]. Discrete choice models allow the composition of available resource units to change prior to each selection of a unit by an animal. This feature was important to our study, because sea ice characteristics can change substantially by winds, currents and the disintegration and northward retreat of sea ice in spring with rising air and water temperatures [34], [35]. We applied discrete choice models to assess the relative importance of the caloric biomass of dominant benthic macroinfauna and sea ice concentration to walruses and to characterize the predicted distribution of selection within our study area.

## Materials and Methods

### 1. General approach

We used discrete choice models [33] to estimate the probability of a walrus selecting a grid cell from a set of available grid cells. For a pair of daily walrus locations, the cells available for selection were identified by a prescribed radius around the walrus location in the first day, and the cell that was selected was the one that contained the walrus location in the second day. Estimates of infaunal caloric biomass and sea ice concentration associated with the available cells (choice set) and selected cell (choice) formed the data set for deriving model estimates. The areas within the St. Lawrence Island polynya that were considered for analysis were delimited by benthic sampling stations occupied during oceanographic field sampling cruises.

### 2. Benthic macroinfauna

**Figure 1:**
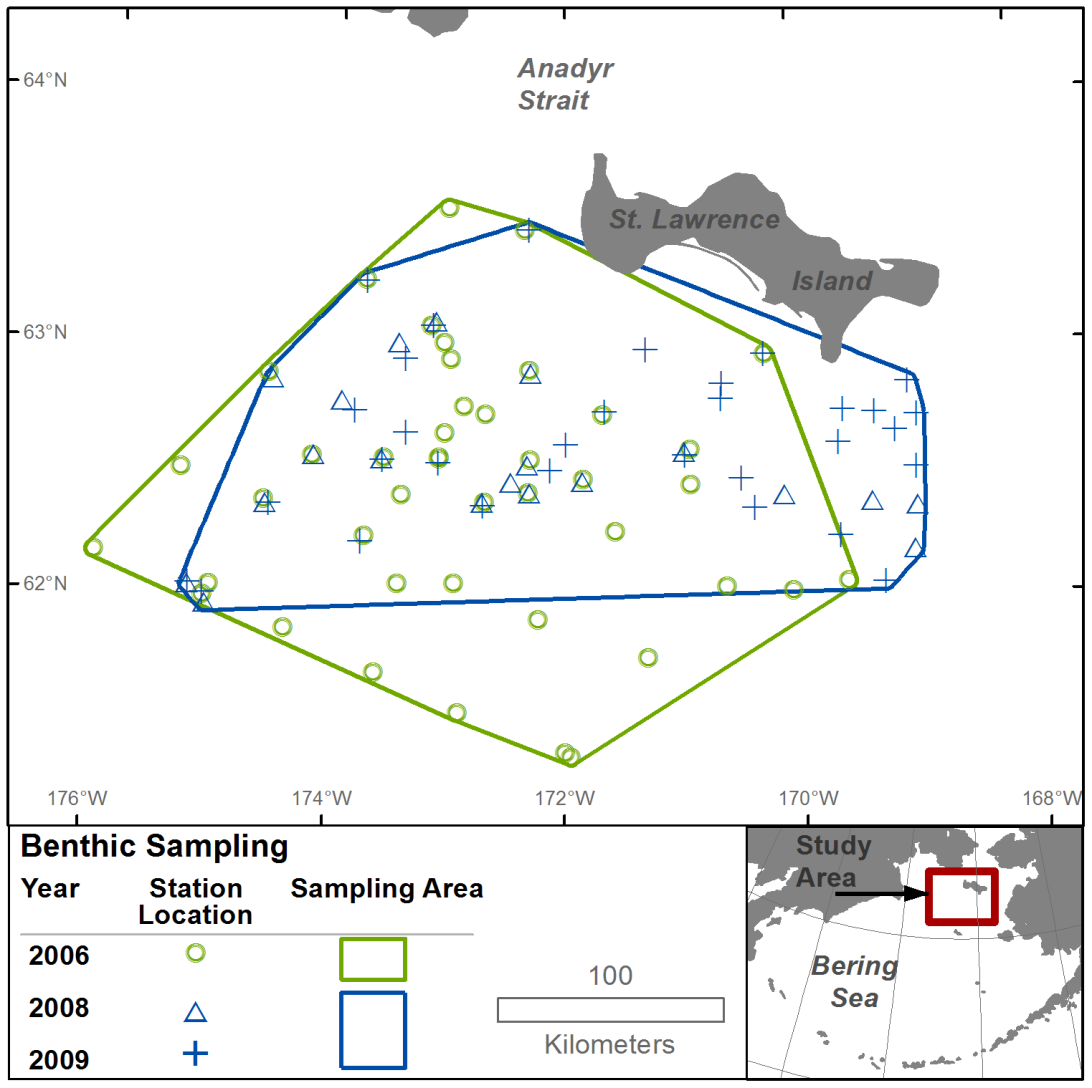
Location of benthic sampling stations. Stations were sampled with a 0.1^2^ van Veen grab in March–June 2006, 2008, and 2009. Stations from 2008 and 2009 were combined in our analysis to represent the distribution of macroinfauna for these years. Sampling areas from the benthic sampling periods (2006 and 2008–2009) were delineated using a minimum convex polygon.

Benthic macroinfauna were collected at stations south of St. Lawrence Island in the northern Bering Sea from the USCGC Healy in March–May in 2006, 2008, and 2009 (Fig. 1). Samples were collected with a 0.1 m^2^ van Veen grab weighted with 32 kg of lead to facilitate penetration into the sediments [36], [37]. Four replicate samples were collected at each station. Some station locations were selected to correspond to the location of samples from past surveys for time-series comparisons in other studies [18], [25], and in 2008 and 2009, some station locations were selected opportunistically to provide additional sampling effort in areas where walruses were encountered. Each grab sample was rinsed with ambient seawater over a 1-mm mesh sieve and retained animals were preserved in 10% hexamethyltetramine buffered seawater formalin and stored in sealed plastic containers prior to identification (further information on standard sampling procedures in [18], [25], [38]). No specific permissions were required for the collection of grab samples at these locations and the collections did not involve endangered or protected species.

Animals were identified to the family level, blotted dry, and then weighed to determine wet mass. We used values of caloric biomass in our analysis to approximate the relative energetic value of potential walrus prey types. Wet mass by taxa within a grab sample was converted to equivalent calories based on conversion values determined from formalin-preserved samples from Stoker [39], except for priapulid worms and sea cucumbers. For priapulids, we used a value similar to those of most polychaete worms, and for sea cucumbers, we used the caloric value in Brawn et al. [40] (Appendix S1). Conversion values ranged from 170 cal/g (Astartidae bivalve) to 1037 cal/g (Lumbrinereidae polychaete).

Caloric density from formalin-preserved samples are higher than fresh-frozen samples for some taxa, particularly bivalves and polychaetes [39], [41], with a recent study indicating a difference of up to 3.3% for similar taxa [42]. However, due to the larger taxonomic diversity we considered, we chose to use the caloric conversions from Stoker [39] and considered the <5% variability between formalin-preserved and frozen samples to be acceptable within the scope of our study. Caloric values were averaged among replicate samples from each sampling station. To minimize geographic distortions during analysis, we transformed the locations of sampling stations with a Lambert equal area projection centered on 168°W and 62°N.

Benthic grab samples from 2006 were collected at 43 stations over an area of 50 260 km^2^; whereas, benthic grab samples from 2008 and 2009 were collected at 20 and 32 stations over an area of 26 891 km^2^ and 39 700 km^2^, respectively. We combined the 2008 and 2009 benthic sampling stations to better estimate the distribution of infauna for these years. The samples from the combined 52 stations in 2008 and 2009 (19 stations were sampled in both years) were collected over an area of 41 795 km^2^ and were used to represent the distribution of macroinfauna in both years. A minimum convex polygon (MCP) around the benthic sampling stations in 2006 and a MCP around the combined stations in 2008 and 2009 formed the areas of analysis for each of those years (these areas collectively are hereafter referred to as the study area). The 2006 and 2008–2009 benthic sampling areas overlapped, but not completely (Fig. 1). For example, some stations in 2006 occupied areas farther south than stations in 2008–2009, and some stations in 2008–2009 occupied areas farther east than stations in 2006.

To estimate the caloric biomass within grid cells in the 2006 and 2008–2009 sampling areas, we interpolated between sampling stations using weighted kernel density estimates. For each benthic sampling area (2006 and 2008–2009) we smoothed station infaunal caloric biomass onto a grid of 2 km×2 km cells across the sampling area. The weighted kernel density estimate for cell *i* was:
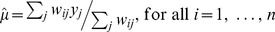
where *y_j_* was the caloric biomass associated with station *j*. Weights were computed as declining functions of distance between the prediction cell and station locations using a multivariate normal kernel. Assuming cell *i* was located at ***θ*** *_i_*, and caloric biomass *j* was located at ***X*** *_j_*, the weight assigned to *y_j_* was computed using the multivariate normal kernel:
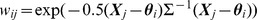
where 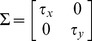 was a matrix whose diagonal values determined the extent of smoothing in the horizontal (*τ_x_*) and vertical (*τ_y_*) directions of the coordinate system being used. To determine *τ_x_* and *τ_y_*, the original Lambert equal area projected coordinate system was rotated through an angle equal to the first principal component of the station locations. This rotation placed the longest axis of the station locations along the horizontal axis of the rotated system, and the shorter axis of the station locations along the vertical axis of the rotated system. Bandwidths 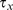 and 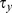 were then chosen in the rotated system using the direct plug-in method [43]. Following computation of 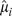 in the rotated space, coordinates were rotated back to the original orientation and plotted (Appendix S2). For each benthic sampling area, we restricted our interpolations to the sampled area defined by the region of the MCP buffered by ½ the mean value of 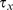 and 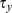.

We determined the contribution of individual taxa at the family level to the total caloric biomass within a sampling area to identify dominant taxa to include as covariates in the discrete choice models. We made this evaluation separately for the 2006 and 2008–2009 benthic sampling areas (Fig. 1). For each sampling area, after ranking the families according to their contribution to total caloric biomass within the area, we calculated the cumulative percent caloric biomass of the families and identified the minimum number of top-ranked families contributing at least 80% of the cumulative caloric biomass. The combined list of these dominant families from each sampling area comprised the taxonomic covariates considered in the resource selection analysis.

### 3. Sea ice

We acquired daily estimates of sea ice concentration based on passive microwave sensor data from the Advanced Microwave Scanning Radiometer - Earth Observing System (AMSR-E) sensor borne by NASA's Aqua satellite and processed to a gridded spatial resolution of 6.25 km [44], http://www.iup.uni-bremen.de:8084/amsredata/asi_daygrid_swath/l1a/n6250, accessed 22 Sep 2011. Sea ice concentration within a 6.25 km×6.25 km pixel can range from open water (i.e., <15% concentration, the concentration at which sea ice can be reliably quantified by passive microwave sensors [45]) to 100% concentration. In addition, we attempted to use weekly (2006) and bi-weekly (2008 and 2009) sea ice stage charts from the U.S. National Ice Center (http://www.natice.noaa.gov/, accessed 22 Sep 2011) in our analysis in the form of digitized geographic polygons with associated ice stage attributes [46], [47]; however, the temporal and spatial resolution of these data were insufficient for adequate model estimation at the resolution of our study (daily selection within a 30-km radius).

### 4. Walrus locations

**Figure 2:**
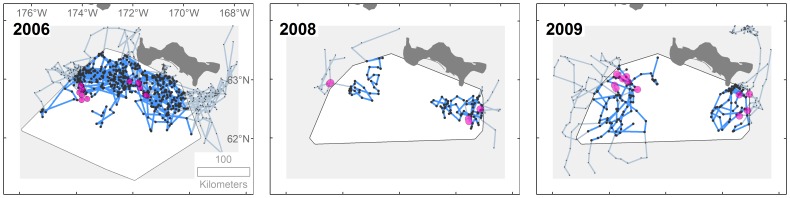
Pacific walrus radio-tracks within benthic sampling areas in 2006 and 2008–2009. Also indicated are radio-tag deployment locations (pink circles) and subsequent daily mean locations (black dots). Movements from the deployment location with durations greater than one day or outside of the benthic sampling area are represented by lighter-colored radio-tracks to provide a larger context of walrus movements.

We attached satellite radio-tags to walruses within the study area in March 2006, 2008, and 2009 (Table 1) using ice-breaking ships to support our field operations. Ice was relatively thick during tag deployments in 2006, making it difficult for the icebreaker to reach the easternmost region of the study area. A fixed-winged aircraft (2006) or a ship-based helicopter (2008 and 2009) provided reconnaissance to locate walrus groups hauled out on the sea ice. We made our final approaches to targeted walrus groups from small boats (2006) or, most often, on foot over the sea ice (all years). Radio-tags were deployed with crossbows from a distance of about 10 m of a targeted walrus (see [5], [34] for more details). We deployed tags opportunistically, as walrus groups were encountered, while attempting to distribute tags as widely as possible among walruses within the eastern and western regions of the study area (Fig. 2). Walrus tagging was conducted under authorization and in accordance of U.S. Fish and Wildlife Service permit number MA801652. The protocol was approved by the USGS Alaska Science Center Institutional Animal Care and Use Committee (approval number 06SOP05).

**Table 1:** Radio-tags attached to Pacific walruses to derive choice sets of available macroinfauna and sea ice concentrations to model resource selection in 2006, 2008, and 2009 in the northern Bering Sea.

Year	Number of walruses				Number of choice sets	Tracking Dates (*n* days that included at least one choice set)
	F	M	U	Total		
2006	26	4	2	32	329	26 Mar–30 Apr (37)
2008	4	2	0	6	75	21 Mar–14 Apr (26)
2009	6	5	0	11	117	18 Mar–25 Apr (39)
Total	36	11	2	49	521	

Geographic locations of tagged walruses were estimated by the Argos location and data collection system [48]. To conserve battery life, transmission duty cycles were set to attempt transmissions for up to 14 hours a day centered on local noon when satellites in the Argos system are most commonly passing overhead. Duty hours were set to attempt transmissions for 14 h starting at 1700 UTC in 2006 and 2008 and for 12 h starting at 1800 UTC in 2009. Transmissions were suspended whenever the radio-tag was submerged. Location estimates were filtered using the Douglas Argos-filter algorithm [49] to exclude implausible locations. The algorithm assesses the plausibility of locations based on spatial redundancy, Argos location quality, maximum rate of movement, and turning angles of successive movements. We set the algorithm to retain (1) all standard class locations, (2) non-standard class locations within 2 km of the previous or subsequent location, and (3) remaining locations based on a distance-angle-rate filter that accepted a maximum walrus speed of 10 km/h and rejected locations at the apex of highly acute angles (RATECOEF = 25, [49]).

Over three times as many radio-tags were deployed in 2006 than in 2008 and 2009 (Table 1). Most radio-tags were deployed on adult females. In each year, walrus locations were obtained within a 25 to 39-d period in March-April (Table 1). Data from walrus locations and associated prey and sea ice concentrations resulted in 521 choice sets that were used in model estimation.

### 5. Model estimation and inference

Discrete choice models were estimated using a stratified Cox proportional hazards model routine as outlined in Manly et al. [1]. Here, the “stratum” of the Cox model consisted of the caloric biomass of benthic macroinfaunal families and sea ice concentration within a choice set of 2 km×2 km grid cells available for selection around the location in the first day of the pair of daily walrus locations. The choice set was identified by a random sample of 300 cells within a 30-km radius (2 827 km^2^ = 707 cells) of the walrus's first location. The 30-km radius was derived from a daily walrus swim speed of 1.3 km/h, which is the average of the 90th percentile daily movement rate of walruses in 2006 in four regions of the northern Bering Sea during the same season as our study (see Figure 5 in [34]). The available cell that was chosen by the walrus was the one that contained the walrus location in the second day of the pair of locations.

Boxplots of covariate values within the choice sets were evaluated in order to assess symmetry, and transformations were applied where necessary to improve symmetry and increase stability of subsequent analyses (Appendix S3). One covariate, the caloric biomass of the bivalve family Mytilidae, was heavily skewed right and was logarithmically transformed.

Collinearity among the macroinfauna and sea ice concentration covariates was assessed with Pearson pairwise correlations. To avoid destabilizing model estimates, a model was restricted to contain only one of a pair of highly correlated covariates.

Models of all possible combinations of main effects were considered, except those that included any combination of correlated covariates. This resulted in fitting 319 models. These models were used to make multimodel inferences with the Akaike's Information Criterion (AIC) [50].

Relative variable importance (RVI) values were used to assess the importance of a predictor variable relative to other variables in the set of models we considered, the larger the value, the more important the variable relative to other variables. RVI was estimated for each variable by summing the Akaike weights across all models in which the variable occurred (Akaike weights indicate the likelihood of a model, given the data, relative to all other models in the set being considered) [50].

We mapped the average probability of walrus selection across the 2 km×2 km cells within the sampling area of each year (2006, 2008, and 2009). For this, for each cell, we summed across the Akaike-weighted predicted probability of walrus selection from all 319 models within each day, and then averaged this daily sum across all days within the year's study period.

## Results

### 1. Benthic Macroinfauna

**Figure 3:**
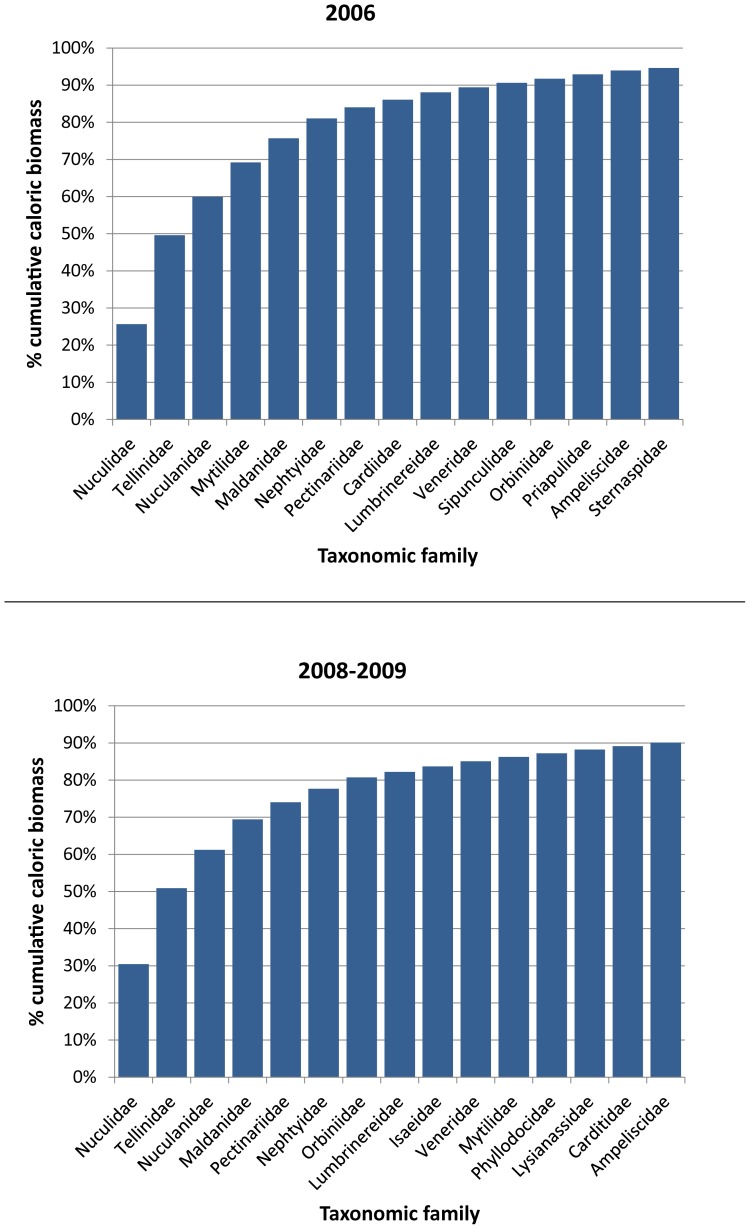
Percent cumulative caloric biomass within benthic sampling areas in 2006 and 2008–2009. Dominant taxa from each sampling period were identified by the minimum number of top-ranked taxa comprising at least 80% of the cumulative caloric biomass (only the first 15 ranked taxa are shown).

The dominant families of macroinfauna, based on interpolated caloric biomass within the sampling areas, were the bivalve families Nuculidae, Tellinidae, Nuculanidae, and Mytilidae, and the polychaete families Maldanidae, Nephtyidae, Pectinariidae, and Orbiniidae (Fig. 3); therefore, these were the infaunal families we considered in our analysis. In both of the benthic sampling areas (2006 and 2008–2009), at least 60% of the total caloric biomass was composed of the nuculid, tellinid, and nuculanid bivalves. The caloric biomass of mytilid bivalves was high in 2006, but not in 2008–2009, and the caloric biomass of pectinariid and orbiniid polychaetes was higher in 2008–2009 than in 2006 (Fig. 3).

### 2. Sea ice

**Figure 4:**
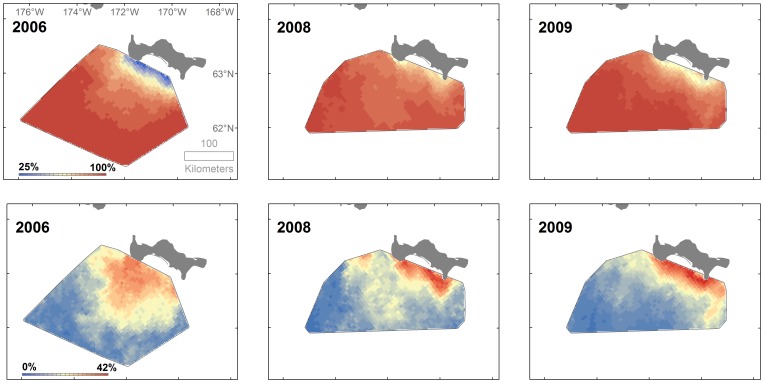
Mean (top row) and sample standard deviation (bottom row) daily sea ice concentration (%). Means and standard deviations were calculated for each 2×2 km cell within the benthic sampling areas in 2006 and 2008–2009 during walrus tracking periods (tracking periods indicated in Table 1).

Sea ice available to walruses for selection within the choice sets over all three years of the study consisted of mostly high concentrations (quartiles: 76%, 93%, and 99%) (Appendix S3). Although mean daily sea ice concentrations were relatively uniform over most of the study area, concentrations can be quite variable within the study period, particularly in the north-northeastern portions of the study area (Fig. 4). This variability is accounted for in our discrete choice modelling approach because sea ice concentration was allowed to vary among choice sets.

**Figure 5:**
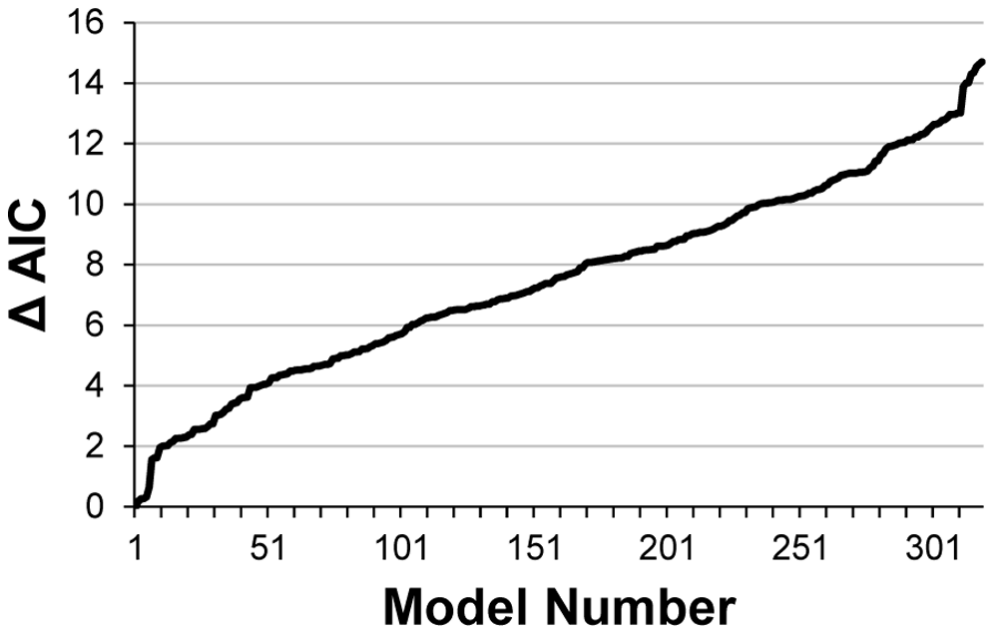
Change in AIC among AIC-ranked models considered in multimodel inferences of walrus resource selection.

### 3. Walrus locations

We radio-tagged 32 walruses in 2006, six walruses in 2008, and 11 walruses in 2009 (Table 1). Seventy-three percent of all walruses tagged were female. Subsequent to tag deployments, most of the tagged walruses in 2006 traveled through the north-central region of the study area; whereas no tagged walruses traveled through this region in 2008 and 2009 (Fig. 2).

### 4. Model estimation and inference

Caloric biomass was highly correlated between the bivalve families Nuculidae and Nuculanidae, and the bivalve family Nuculidae and polychaete family Pectinariidae (>0.60 Pearson correlation, Table 2), and therefore, models were restricted to contain only one of the correlated covariates, but not both. We considered inclusion of a quadratic term for the sea ice concentration variable in the models; however, its squared term was highly correlated with its linear term, therefore models were restricted to include only the linear term.

**Table 2:** Correlations among covariates used in models fitted to estimate Pacific walrus resource selection in the northern Bering Sea (correlations >0.60 in bold).

	Ice Concentration	Nuculanidae	Tellinidae	Nuculidae	Mytilidae	Maldanidae	Nephtyidae	Pectinariidae	Orbiniidae
**Ice Concentration**	1.00	0.20	−0.24	0.17	0.08	0.20	−0.08	0.16	0.01
**Nuculanidae (B)**		1.00	0.07	**0.79**	0.54	0.41	−0.14	0.60	0.05
**Tellinidae (B)**			1.00	0.24	−0.12	−0.11	0.00	0.18	−0.04
**Nuculidae (B)**				1.00	0.55	0.19	−0.39	**0.79**	0.08
**Mytilidae (B)**					1.00	0.21	0.02	0.43	0.14
**Maldanidae (P)**						1.00	0.13	0.22	0.23
**Nephtyidae (P)**							1.00	−0.40	0.09
**Pectinariidae (P)**								1.00	0.26
**Orbiniidae (P)**									1.00

B = Bivalvia, P = Polychaeta.

Differences in AIC values among models, ordered from smallest to largest (Fig. 5), indicated that each model beyond the first ten models explained little additional variation in the data. The greatest differences in AIC values occurred among the first six models; however, even these differences were small. Because of this lack of a clear single best model, we made inferences from the full set of models fitted to the data (multimodel inferences), which is likely to be more robust than inferences from a single best model [50].

The high RVI values for Tellinidae and Orbiniidae (Table 3), and the fact that they were the only covariates that occurred in all of the first six models (Table 4), suggests that they were the two most important covariates in predicting walrus site selection. RVI values suggest that the Nephtyidae, ice concentration, and Nuculanidae covariates were moderately important in walrus site selection and that the Mytilidae, Maldanidae, Pectinariidae, and Nuculidae covariates were negligibly important in walrus site selection. The direction of covariate coefficients across the set of models (Table 3) suggest that walruses selected for areas associated with high caloric biomass of tellinid bivalves, low caloric biomass of orbiniid and nephtyid polychaetes and nuculanid bivalves, and low sea ice concentration.

**Table 3:** Relative variable importance and direction of variable coefficients among 319 models fitted to estimate Pacific walrus resource selection in the northern Bering Sea.

Variable	Relative variable importance	Direction of relationship^a^
Tellinidae	0.92	+
Orbiniidae	0.75	−
Nephtyidae	0.64	−
Ice Concentration	0.56	−
Nuculanidae	0.53	−
Mytilidae	0.31	+
Maldanidae	0.27	−
Pectinariidae	0.25	−
Nuculidae	0.23	−

a Estimated from model average coefficients [50].

**Table 4:** Ten highest-ranked models among 319 models fitted to estimate Pacific walrus resource selection in the northern Bering Sea.

Rank	Model	AIC	AIC Difference
1	IceConc Nuculanidae Tellinidae Nephtyidae Orbiniidae	5849.73	0.00
2	IceConc Tellinidae Nuculidae Nephtyidae Orbiniidae	5849.92	0.19
3	IceConc Nuculanidae Tellinidae Orbiniidae	5850.00	0.27
4	Nuculanidae Tellinidae Nephtyidae Orbiniidae	5850.00	0.27
5	Nuculanidae Tellinidae Orbiniidae	5850.06	0.33
6	Tellinidae Nuculidae Nephtyidae Orbiniidae	5850.37	0.64
7	IceConc Nuculanidae Tellinidae Nephtyidae Pectinariidae Orbiniidae	5851.29	1.56
8	Nuculanidae Tellinidae Nephtyidae Pectinariidae Orbiniidae	5851.35	1.62
9	IceConc Nuculanidae Tellinidae Mytilidae Nephtyidae Orbiniidae	5851.36	1.63
10	IceConc Nuculanidae Tellinidae Mytilidae Orbiniidae	5851.69	1.96

**Figure 6:**
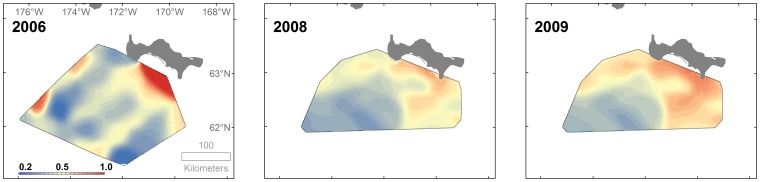
Average probability of walrus resource selection within benthic sampling areas in 2006 and 2008–2009.

The distribution of average predicted walrus site selection was similar in 2008 and 2009, and selection in both years was somewhat different than in 2006 (Fig. 6). In general, average predicted selection in all three years was highest in the western, northern, and northeastern parts of our study area, which extended over areas of 50 to 100 km.

## Discussion

Members of eight families of benthic infauna comprised over 80% of the total estimated caloric biomass in our study area. However, the only family of infauna that was positively associated with walrus site selection was the bivalve family Tellinidae. Caloric biomass within Tellinidae was primarily represented by the shallow-dwelling bivalves *Macoma calcarea* and *M. moesta* (Appendix S1). Tellinidae, and the bivalve families Nuculidae and Nuculanidae, were the highest contributors to infaunal caloric biomass (>60% cumulative caloric biomass). Although shifts in species dominance have occurred among members of these families over the past few decades (1980s through 2000s) [20], [31], these families have been among the dominant taxa of infaunal groups in this area since at least the late 1970s [30], [38], [39]. The relative importance of Nuculidae and Nuculanidae to walrus selection may have been partially obscured in our analysis, because they were not included together in the same model, nor was Nuculidae and the polychaete family Pectinariidae included in the same model, due to their collinearities.

Walrus selection for sites with high tellinid bivalve caloric biomass is inconsistent with the frequencies of prey items found among fresh walrus stomachs collected across a wide area of the Bering Sea in past decades (1954–1991) [22], but it is unclear how many (if any) of those stomachs were collected from walruses within our study area (south of St. Lawrence Island). Although bivalves were the most prevalent prey items in the stomachs examined by Sheffield and Grebmeier [22], few to no stomachs contained tellinid bivalves; instead, they most frequently contained the larger-bodied shallow-dwelling bivalves *Serripes* (F. Cardiidae) and deep-dwelling bivalves *Mya* (F. Myidae). Dissimilarities in the frequency of infaunal taxa found in walrus stomachs and of taxa from benthic grab samples have been noted in past studies [30]. Gastropods occurred in 83% of the walrus stomachs [22], but gastropods were not included in our study because they are highly motile epifauna, which are not adequately sampled by the van Veen grab. Fish, birds, parts of seals, and other non-infaunal taxa were represented in a small percentage of the stomachs examined by Sheffield and Grebmeier [22] and it is possible that at times these items make up a significant portion of the caloric intake of some individuals.

Polychaete worms were also prevalent in the walrus stomachs (78% of stomachs) of past range-wide studies in the Bering Sea [22], but we found no evidence that the caloric biomass of polychaetes were positively associated with walrus site selection in our study area. On the contrary, we found that orbiniid and nephtyid polychaetes were negatively associated with site selection. The distribution of benthic communities, which are often characterized by high species dominance, is largely influenced by hydrography and the distribution of sediment grain sizes [20], [25]. Orbiniid and nephtyid polychaetes were not likely avoided by walruses, but were probably less preferred, so that walrus site selection in favor of communities dominated by tellinid bivalves resulted in an apparent selection for sites where the caloric biomass of these polychaetes was low relative to their caloric biomass at other available sites.

Walruses feed as deep as about 30 cm in bottom sediments containing deep-dwelling bivalves [51], but the limited 10–15 cm sampling depth of the van Veen grab cannot penetrate to those depths and adequately sample deep-dwelling bivalve prey, such as *Spisula* (F. Mactridae) and *Mya* [30], [38], [52]. Therefore, the available caloric biomass from deep-dwelling taxa may have been underrepresented in our study; although, significant recruitment of small individuals of *Spisula* and *Mya* into the upper layers of sediment was not indicated by the contents of our benthic samples. The reduced penetration of the van Veen grab in sandy sediments also limited adequate sampling in nearshore areas just south of St. Lawrence Island [25].

Areas with high average predicted walrus site selection (western, northern, and northeastern parts of our study area) generally coincided with areas of high organic carbon input from higher productivity associated with Anadyr water flowing eastward immediately south of St. Lawrence Island. Ecosystem studies in this region indicate early spring ice edge primary production in the area south of the St. Lawrence Island polynya with a high degree of sedimentation to the benthos [25], [26], [27], [28]. The area west (and south) of the polynya is high in benthic biomass due to high export of production to the benthos in this region [18], [20]. The slight dissimilarities in average predicted site selection between 2008 and 2009 (Fig. 6) must have been entirely due to differences in daily sea ice concentration, because the same estimates of caloric biomass of macroinfaunal taxa were used in the predictions for both of these years; in contrast, different ice concentrations and macroinfaunal caloric biomass estimates were used to predict selection in 2006.

Walruses selected lower ice concentrations within the mostly high concentrations available to them in the choice sets (quartiles: 76%, 93%, and 99%). The need for sufficient ice concentration and thickness to rest upon, and simultaneously, for open water that allows access to feeding on the underlying benthic fauna, likely influences this pattern. The location of low ice concentration in early spring is subject to regional weather events, wind direction, and current patterns, all combining to influence resource partitioning for predator access to prey patches. During our studies in March–April, sea ice was thinner in 2008 and 2009 than in 2006 (unpublished plots of ice thickness using data from the U.S. National Ice Center, http://www.natice.noaa.gov/, accessed 22 Sep 2011), which coincided with the lack of movement of tagged walruses across the northern part of our study area in those years (Fig. 2). However, in 2006, a year when it was relatively cold and thick ice prevailed, most of the tagged walruses moved throughout the northern region. Ice concentration can vary substantially within seasons and among years and has been observed to influence the location of other animals and their access to resources in the St. Lawrence Island polynya, such as the spectacled eider (*Somateria fischeri*) sea duck and its access to bivalve prey [28], [53].

At the time of year of our study (March–April), walruses would have been in the study area since the beginning of winter when they selected the area of the St. Lawrence Island polynya after migrating south from the Chukchi Sea and into their winter home range. Walruses are not able to penetrate and effectively utilize areas with very high ice concentrations [7], such as current winter conditions in the Chukchi Sea where ice concentrations of >90% commonly occur [8]. The finer-level temporal and spatial selection addressed in our study reflects walrus site selection within the context of walruses having already selected a wintering area that presumably possesses a combination of attributes of sea ice and benthic prey that best meets their needs for over-wintering. The predictability of the recurrence of the St. Lawrence Island polynya each winter with its features of unconsolidated ice and access to areas of high productivity and benthic biomass may adequately explain the seasonal selection of this area by walruses (and other marine mammals and seabirds) [54].

Our study suggests that walruses primarily selected sites associated with high tellinid bivalve caloric biomass in the St. Lawrence Island polynya. Although the van Veen grab was unable to sample older individuals of deep-dwelling bivalves, the absence of substantial biomass of these taxa in the surface sediments sampled by the van Veen grab suggests recruitment of these bivalves into deep sediments may be minimal. Since differences in caloric density among bivalve taxa were not great, bivalve selection may have been partially related to the size of available clams. The concentration of sea ice was also an important variable for walrus site selection and is likely related to the need for walruses to have access to sufficient sea ice for hauling out to rest, while simultaneously having access to enough open water to breath at the surface between foraging dives to underlying benthic prey. The fine-level temporal and spatial walrus selection we addressed in our study (daily selection at <30-km spatial scale) was probably sufficiently constrained by our inability to consider other fine-scale sea ice metrics (e.g. thickness) and the limitations imposed by interpolating caloric biomass values between relatively distant benthic sampling stations (nearest neighbor 2006 quartiles: 11 km, 25 km, and 35 km; 2008–2009 quartiles: 12 km, 15 km, and 22 km).

Increases in air and sea temperatures and a reduction of 42% of spring sea ice extent are projected to occur in the Bering Sea by 2050 [9]. These physical properties are important driving factors for biological processes and have a large influence on the productivity of benthic infauna in the northern Bering Sea. In addition, the extent and duration of a unique cold pool resulting from winter ice formation within the region of the St. Lawrence Island polynya probably enhances the standing stock of benthic walrus prey by excluding certain fish and other epibenthic predators of the benthos [20]. Projected decreases in sea ice concentration and extent in the St. Lawrence Island polynya and the potential for a concomitant decline of bivalves in the region could result in a northward shift in the wintering grounds of walruses in the northern Bering Sea. Further walrus resource selection studies throughout the annual range of the population would enhance our ability to forecast the response of walruses to future changes in the Arctic ecosystem.

## Supporting Information

Appendix S1 **Mean station caloric biomass of macroinfauna.** Wet mass infauna was sampled with a 0.1 m^2^ van Veen grab in March–June 2006, 2008, and 2009 (see Figure 1). See text for reference to caloric equivalents of wet mass. Stations from 2008 and 2009 were combined in our analysis to represent the distribution of macroinfauna for these years.
(PDF)

Appendix S2 **Interpolated caloric biomass of dominant benthic macroinfauna within benthic sampling areas.**
(PDF)

Appendix S3 **Macroinfaunal caloric biomass (Kcal/m^2^) and sea ice concentration (%) available within walrus choice sets.** Choice sets comprised data from March–April in 2006, 2008, and 2009 and were used to estimate walrus selection (Mytilidae was log-transformed for analysis). Boxes indicate the 25th, 50th, and 75th quartiles and whisker caps indicate range.
(PDF)
