# Supplementary material for: Pacific Walrus (Odobenus rosmarus divergens) Resource Selection in the Northern Bering Sea
Source: PLoS One. 2014 Apr 9;9(4):e93035. doi: 10.1371/journal.pone.0093035 (PMC3981674; doi:10.1371/journal.pone.0093035)
Supplement: Appendix S1 — Mean station caloric biomass of macroinfauna. Wet mass infauna was sampled with a 0.1 m2 van Veen grab in March–June 2006, 2008, and 2009 (see Figure 1). See text for reference to caloric equivalents of wet mass. Stations from 2008 and 2009 were combined in our analysis to represent the distribution of macroinfauna for these years. (PDF) [file pone.0093035.s001.pdf]

		Mean station caloric biomass (Cal/m^2)		
**Taxon**	**Cal/gwm**	**2006**	**2008**	**2009**
Polychaeta	715	0	100	40
Polynoidae	715	40	50	40
Sigalionidae	715	10	40	20
Phyllodocidae	715	40	170	130
Nephtyidae	779	610	500	430
Glyceridae	715		30	80
Goniadidae	715	20	10	20
Onuphidae	715	100	110	130
Lumbrinereidae	1037	280	320	180
Orbiniidae	715	150	220	360
Spionidae	715	0	0	30
Magelonidae	715		60	0
Cirratulidae	715	20	50	90
Flabelligeridae	523	40	30	20
Scalibregmidae	715	10	10	30
Opheliidae	715	120	40	190
Sternaspidae	410	260	170	110
Capitellidae	715	50	50	70
Maldanidae	754	850	1220	760
Oweniidae	715	10	0	30
Pectinariidae	715	550	960	400

Ampharetidae	762	80	0	80
Terebellidae	715	210	230	50
Trichobranchidae	715	110	10	110
Sabellidae	815	10	10	30
Bivalvia	327			140
Nuculidae	461	390	610	0
Ennucula tenuis	461	3900	4300	3330
Nuculanidae	233		10	40
Nuculana radiata	233	1540	2170	1160
Nuculana pernula	233			240
Nuculana sp	233		140	
Portlandia arctica	233			290
Yoldia sp.	551	220	280	290
Mytilidae	327	1350	40	550
Astartidae	170			60
Carditidae	327	230		1180
Lucinidae	327		0	20
Thyasiridae	327	10	20	30
Cardiidae	327		200	20
Clinocardium sp.	327	300	550	30
Serripes groenlandicus	438	1880	1490	140
Veneridae	327	360	170	570
Mactridae	327		20	

Tellinidae	433	0	700	490
Macoma calcarea	389	3040	1370	860
Tellina lutea	433		360	30
Macoma moesta	389	210	720	990
Tellina sp.	433			70
Macoma sp.	389		160	320
Myidae	327	20		0
Hiatellidae	327			20
Periplomatidae	327	300		
Thraciidae	327	0		30
Gastropoda				
Trochidae	692	20	290	60
Cylichnidae	692	30	60	70
Amphipoda	814	0	20	10
Ampeliscidae	814		0	20
Ampelisca sp.	814	190	80	60
Byblis sp.	814	190	140	70
Aoridae	814	20	460	
Corophiidae	814	10	20	20
Gammaridae	814	0	50	130
Haustoriidae	814	90	110	150
Isaeidae	814	60	90	240
Lysianassidae	814	70	50	90

Oedicerotidae	814	10	20	10
Phoxocephalidae	814	10	30	60
Synopiidae	814		20	0
Melitidae	814	140		120
Sipuncula				
Sipunculidae	544	380	30	50
Priapula				
Priapulidae	710	210	190	30
Holothuroidea	224			50
Synaptidae	224	20	20	60
