# Supplementary figures and images for: Pacific Walrus (Odobenus rosmarus divergens) Resource Selection in the Northern Bering Sea
Source: PLoS One. 2014 Apr 9;9(4):e93035. doi: 10.1371/journal.pone.0093035 (PMC3981674; doi:10.1371/journal.pone.0093035)

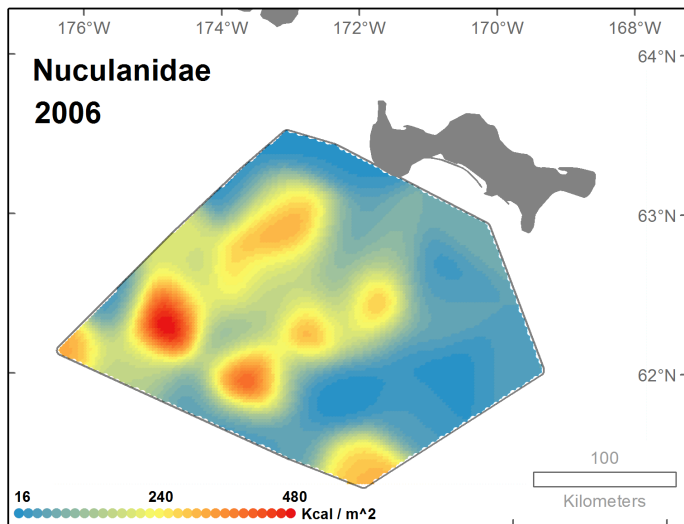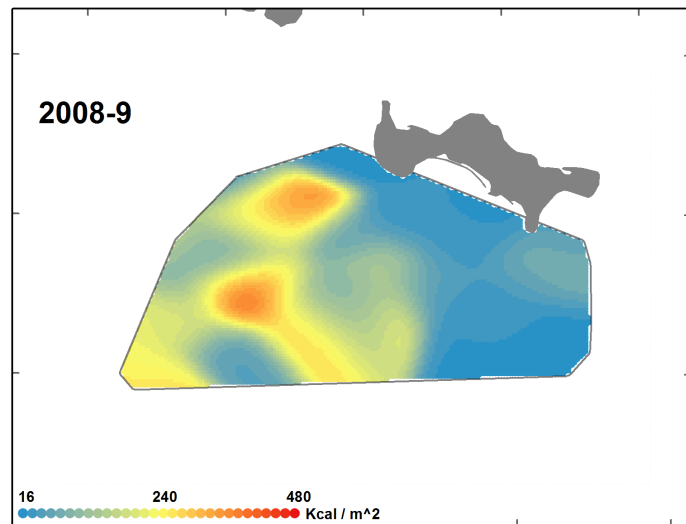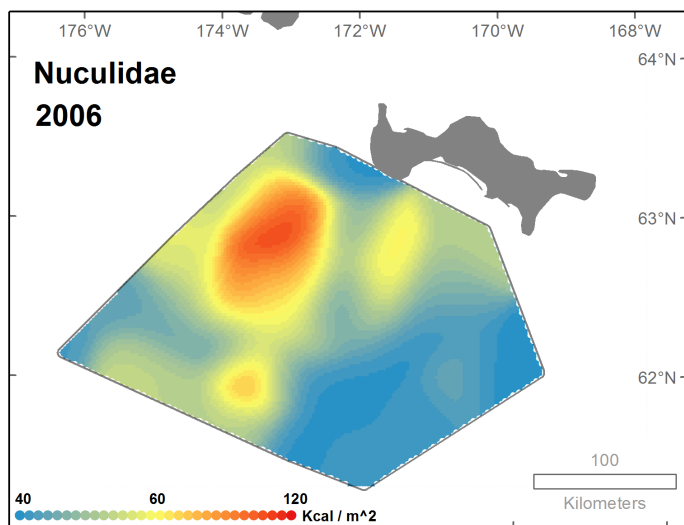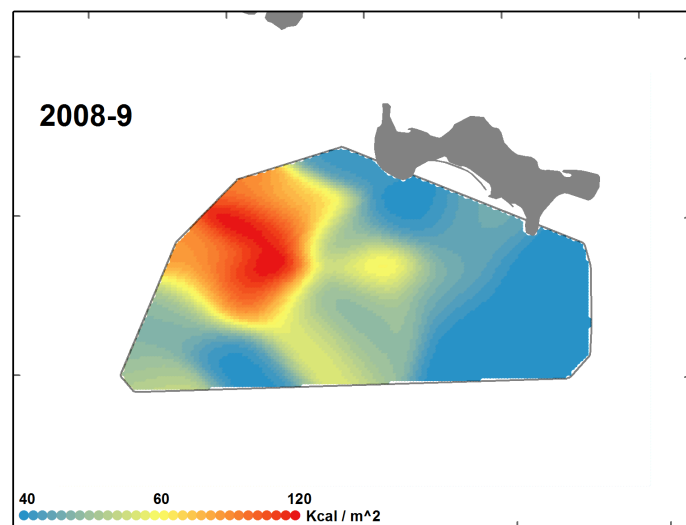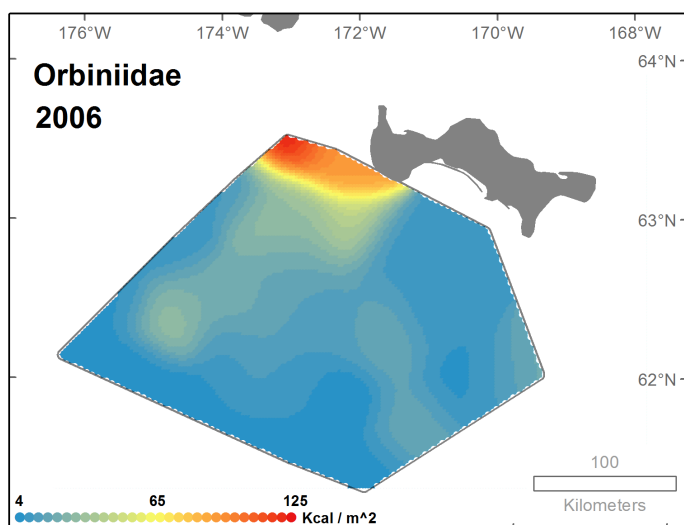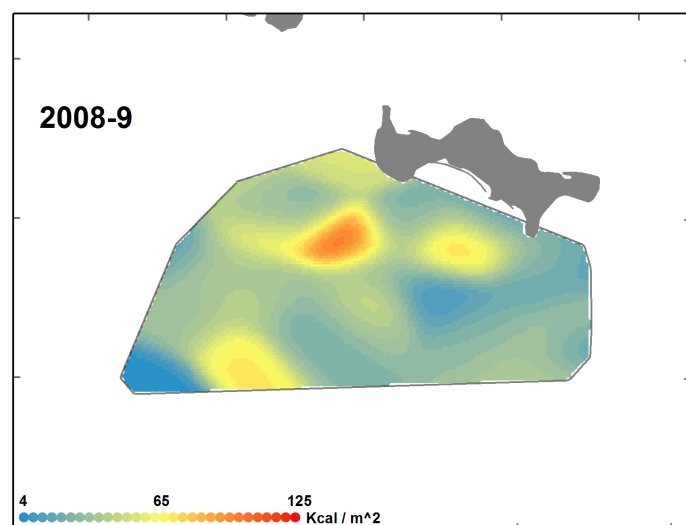

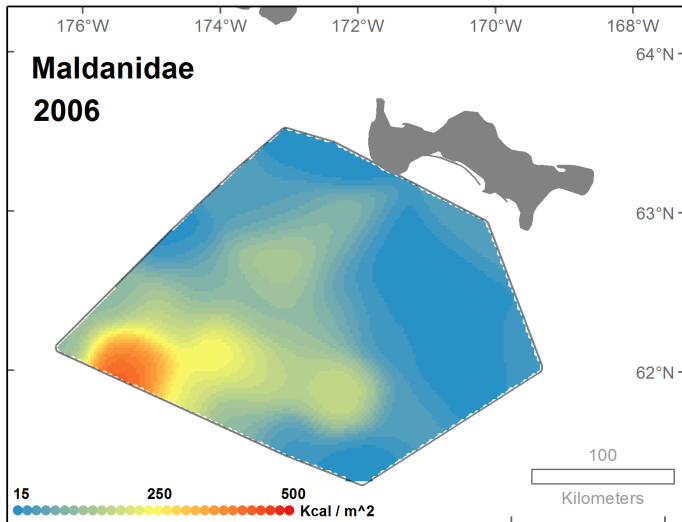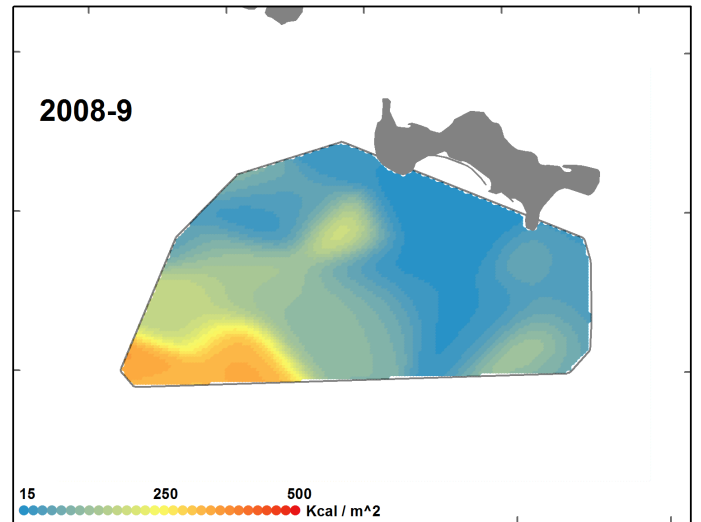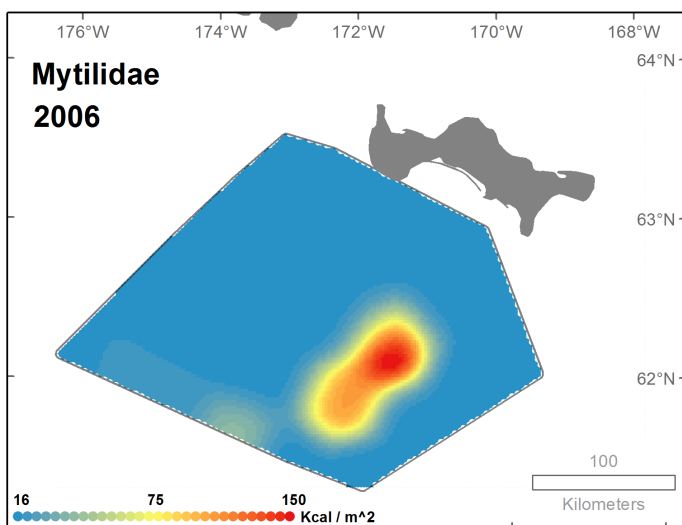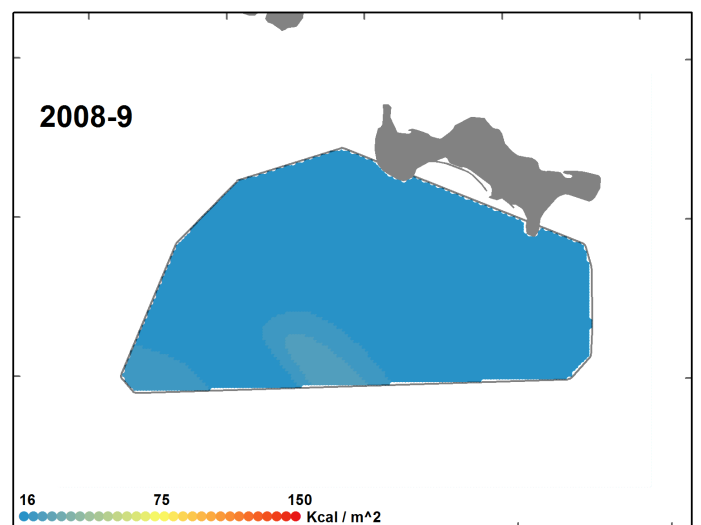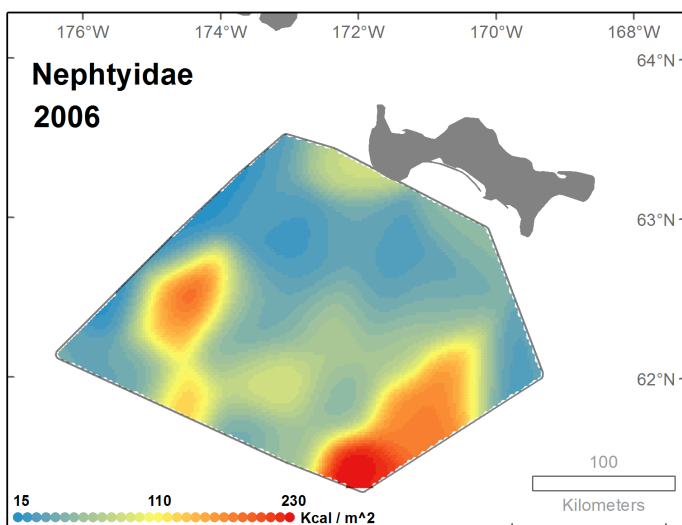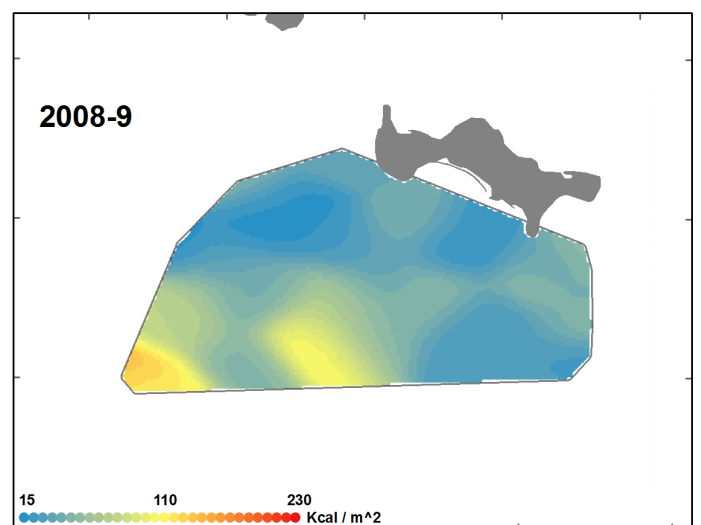

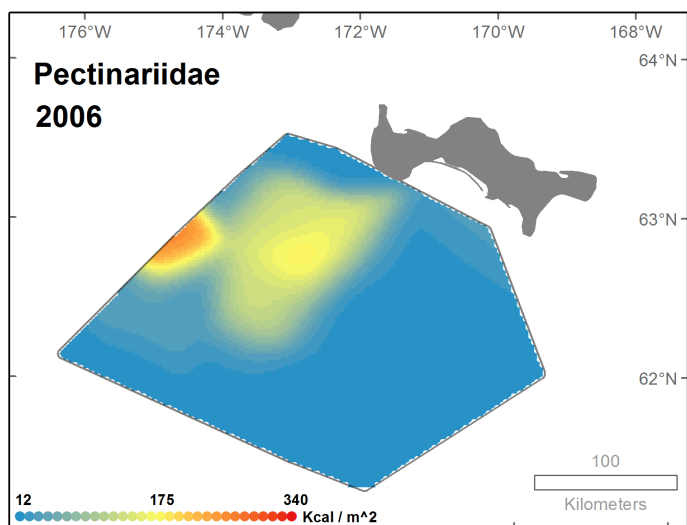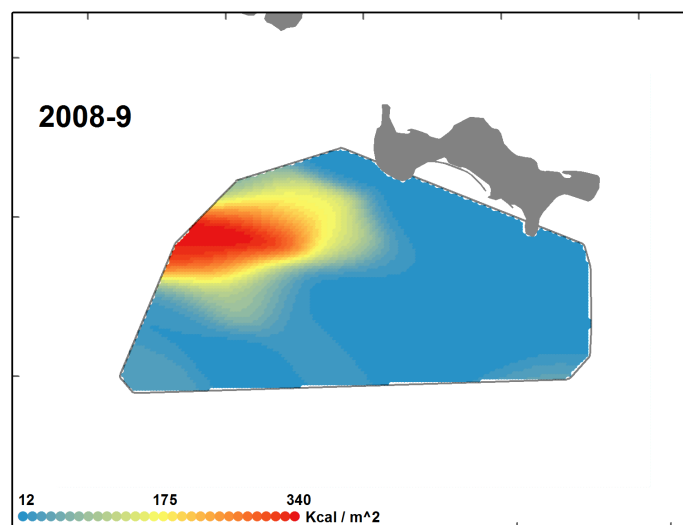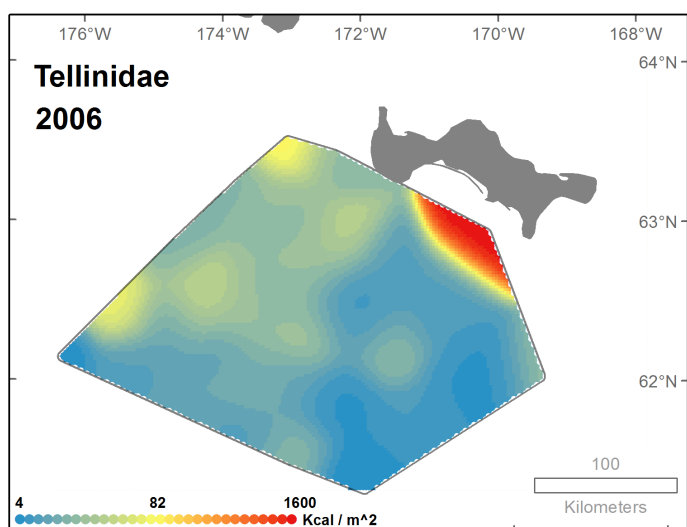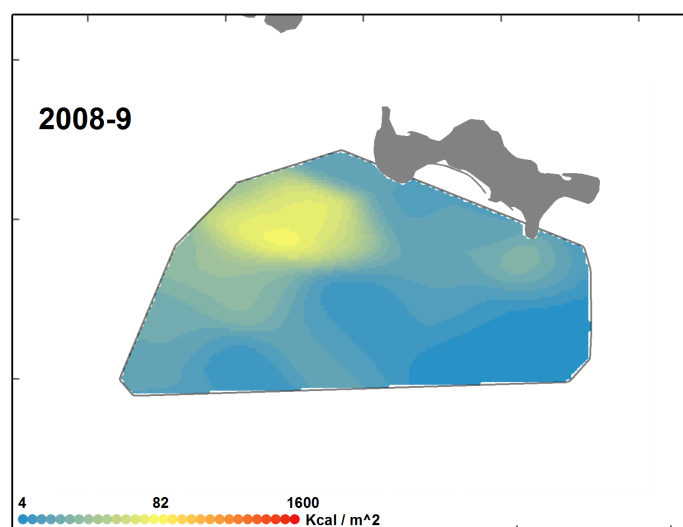

Supplement: Appendix S2 — Interpolated caloric biomass of dominant benthic macroinfauna within benthic sampling areas. (PDF) [file pone.0093035.s002.pdf]
