# Supplementary material for: Pacific Walrus (Odobenus rosmarus divergens) Resource Selection in the Northern Bering Sea
Source: PLoS One. 2014 Apr 9;9(4):e93035. doi: 10.1371/journal.pone.0093035 (PMC3981674; doi:10.1371/journal.pone.0093035)
Supplement: Appendix S3 — Macroinfaunal caloric biomass (Kcal/m2) and sea ice concentration (%) available within walrus choice sets. Choice sets comprised data from March–April in 2006, 2008, and 2009 and were used to estimate walrus selection (Mytilidae was log-transformed for analysis). Boxes indicate the 25th, 50th, and 75th quartiles and whisker caps indicate range. (PDF) [file pone.0093035.s003.pdf]

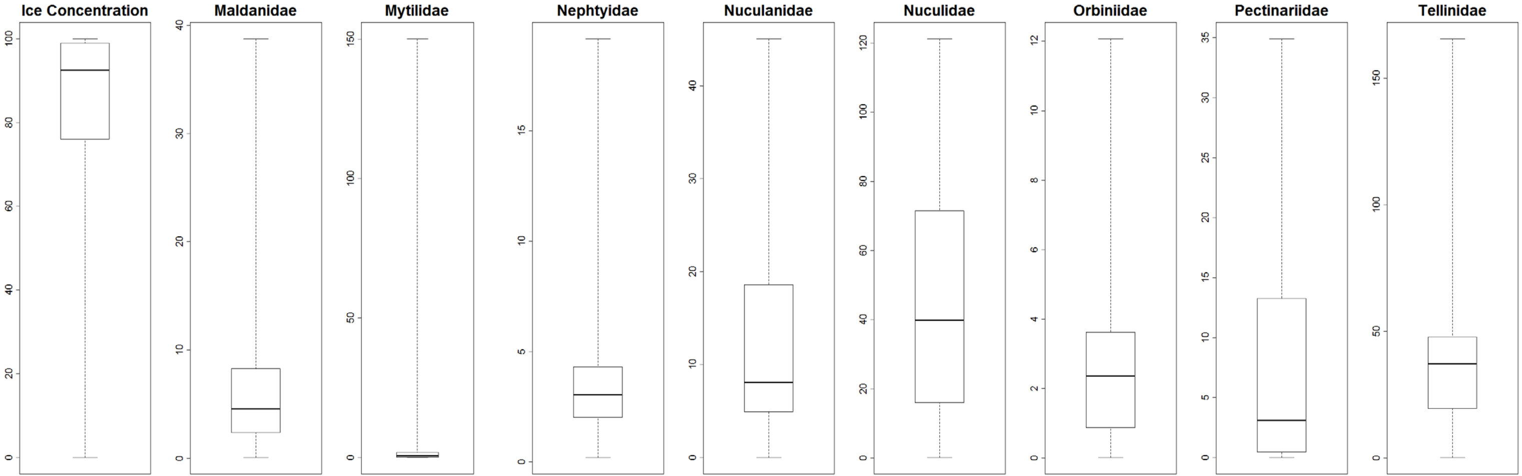

Ice Concentration
Maldanidae
Mytilidae
Nephtyidae
Nuculanidae
Nuculidae
Orbiniidae
Pectinariidae
Tellinidae
